# Cooling Drinks with Simple Recipes

**Published:** 1912-08-03

**Authors:** 


### INSTITUTIONAL HOUSEKEEPING.

### Cooling Drinks with Simple Recipes.

The matter of beverages for all inmates of the institution, staff as well as patients, is often much neglected. Over-much tea and coffee are consumed because no other choice is offered, unless indeed it be "flat" and often tepid water. A few simple recipes for cooling drinks will be appreciated at this season.

Two excellent beverages, the recipes for which were originally prepared for a prominent worker for the Church of England Temperance Society, are much used where large numbers are to be provided with cooling drink, and may prove welcome in some institutions. They are prepared as follows:—

### Hopkos.

Put six quarts of water on the fire, add to it three-quarters of an ounce of hops and half an ounce of dried ginger well bruised by beating it with a rolling-pin. Boil these for half an hour. Then add three-quarters of a pound of Demerara sugar and boil again for ten minutes. Strain off the liquid and bottle whilst still hot, or, better still, put it in a clean cask. It is ready to use when quite cold. If kept, store it in a cool place. Costs about threepence per gallon.

### Stokos.

Put into a clean saucepan 4 oz. of fine oatmeal, 6 oz. of moist sugar, and two tablespoonfuls of lime-juice. Mix these smoothly and gradually with enough warm water to make a thin smooth mixture. Then pour on four quarts of boiling water. Stir well, leave until cold, and then strain. It may be given hot in cold weather. Both these beverages may be improved by adding a liberal flavouring of fresh lemon-juice and chipped-off lemon rinds; or by adding extra lime-juice or ginger, to taste. If cooled with ice before serving they will be found specially refreshing.

### Home-made Gingerbeer.

Put the thinly pared rinds and strained juice of three lemons into a large earthenware jar or bowl. Add to these 3 lb. of loaf sugar. Mix in gradually 2 oz. of cream of tartar, and quickly pour in two gallons of boiling water. Add about 2 oz. of bruised dried ginger, cover the pan with a thick cloth, and leave it until the liquid is tepid. Make a fairly thick slice of toast, spread over it either a tablespoonful of brewer's yeast or an ounce of compressed yeast. Lay this toast in the liquid to start fermentation. Leave for twenty-four hours, then strain off, bottle, cork down, and it will be fit to use in three or four days.

### Barley Water: Clear and Thick.

This delicious beverage is no longer reserved for invalids, but is now a regular item in many of the best clubs and restaurants. Make it like this: Put 3 oz. of pearl barley in a clean saucepan, add cold water to cover the barley and heat slowly to boiling-point. Boil for a minute; then strain off and throw away the water. This blanches the barley and improves its colour and flavour. Next turn the barley into a clean jug, add to it the very thinly pared rind of one lemon and about four lumps of sugar. Pour on to these one pint of boiling water, cover the jug tightly, and leave till cold. Then strain off the liquid into a glass jug and set on ice if possible till needed. More or less of lemon peel can be added to taste.

For thick barley water use the same ingredients, but after the blanching boil the ingredients gently until the mixture is about the consistency of thin cream. Remember barley water does not keep well.

### Orangeade.

This is most refreshing. Take two oranges; wipe them, chip off the rinds as thinly as possible, and put them into a clean jug. Strip off all the white pith from the peeled fruit, slice them thinly, remove pips, and put the slices also in the jug. Add about an ounce of loaf sugar, pour over one and a half pint of freshly boiling water, cover the jug, and let it stand until its contents are cold. Then strain off the liquid and serve it cold—iced if possible.

### Lemonade.

This is made in the same way as orangeade. These infusions are far nicer and more economical than when the juice is merely squeezed into water, as the essential oil in the tiny cells in the rinds is a valuable ingredient, which should not be wasted.

### King's Cup.

Put the thinly pared rind and strained juice of a lemon in a jug. Add about two inches of whole-dried ginger, first bruising it well by beating it with a rolling-pin; also 1 oz. of loaf sugar and two unpeeled sliced apples. Pour over these a quart of boiling water. Cover the jug and leave until the liquid is cold; then strain it off and use. For special occasions add a glass of sherry just at the last.

### Lemon Froth.

Strain the juice of a lemon into a glass jug, add a pint of cold water and 1 oz. of castor sugar. Beat the white of an egg to a stiff froth and beat it well into the mixture. Serve at once as cold as possible. You will note that owing to the white of egg this drink is nourishing as well as refreshing. This same mixture is delicious if half frozen. The value of all these drinks is doubled by the admixture of a little ice.

### Drinking Water.

The heads of establishments would do well to see that their inmates are served with cool refreshing water, sent to table in brightly polished glass jugs to be drank from clear, reasonably thin tumblers. Where it is desirable to boil the drinking water the flat taste which is so objectionable must be removed by pouring the water backwards and forwards from one vessel to another, so as to re-aerate it and bring back the sparkle. It should then, in hot weather, be poured into a vessel which can be stood in another containing the coldest water obtainable and a handful or two of salt. In establishments where ice is in constant use or made on the premises it is difficult to see why the small amount that would be required to chill the drinking water cannot be easily obtained. The fact is that English people do not attach enough importance to this.

Even at elaborate dinner parties, with a choice of costly wines, the guest who prefers cold water is often treated to a glass of lukewarm stuff tasting of the tap. No item in the menu needs more urgently the unremitting attention of the housekeeper, for without ceaseless vigilance the water will be permitted to grow stale in the jugs from one day to another, to stand in the sun, or accumulate the flavour of whatever happens to be near it.

